# National impact of ICD-11 stroke reclassification on projected incidence across the United Kingdom

**DOI:** 10.1093/eurpub/ckag133

**Published:** 2026-07-22

**Authors:** Camila Pantoja-Ruiz, Eva S Emmett, Evelyn Lim, Wasana Kalansooriya, Sophie Rowland-Coomber, Svetlana Mamac, Tayla Schaapveld, Abdel Douiri, Julia Fox-Rushby, Yanzhong Wang, Ajay Bhalla, C D A Wolfe, Matthew D L O’Connell, Iain J Marshall

**Affiliations:** Department of Population Health Sciences, School of Life Course & Population Sciences, King’s College London, London, United Kingdom; Department of Population Health Sciences, School of Life Course & Population Sciences, King’s College London, London, United Kingdom; Department of Population Health Sciences, School of Life Course & Population Sciences, King’s College London, London, United Kingdom; Department of Population Health Sciences, School of Life Course & Population Sciences, King’s College London, London, United Kingdom; Department of Population Health Sciences, School of Life Course & Population Sciences, King’s College London, London, United Kingdom; Department of Population Health Sciences, School of Life Course & Population Sciences, King’s College London, London, United Kingdom; Department of Population Health Sciences, School of Life Course & Population Sciences, King’s College London, London, United Kingdom; Department of Population Health Sciences, School of Life Course & Population Sciences, King’s College London, London, United Kingdom; Department of Population Health Sciences, School of Life Course & Population Sciences, King’s College London, London, United Kingdom; Department of Population Health Sciences, School of Life Course & Population Sciences, King’s College London, London, United Kingdom; Department of Population Health Sciences, School of Life Course & Population Sciences, King’s College London, London, United Kingdom; Department of Ageing and Health and Stroke, Guy’s and St Thomas’ NHS Foundation Trust, London, United Kingdom; Department of Population Health Sciences, School of Life Course & Population Sciences, King’s College London, London, United Kingdom; Department of Population Health Sciences, School of Life Course & Population Sciences, King’s College London, London, United Kingdom; Department of Population Health Sciences, School of Life Course & Population Sciences, King’s College London, London, United Kingdom

## Abstract

The ICD-11 stroke definition expands stroke diagnosis by including neuroimaging-confirmed brain lesions regardless of symptom duration. Using directly age- and sex-standardized rates from the South London Stroke Register applied to UK Census 2021/2022 populations, we projected that ICD-11 to increase UK stroke incidence by ∼4.2%, yielding an estimated 2771 additional cases annually (England +2313; Scotland +241; Wales +142; Northern Ireland +75). Ethnicity-adjusted estimates suggested a 6.2% increase for England and Wales. These projections are scenario estimates that assume South London rates apply uniformly across the UK, their central limitation, and their realization will depend on local imaging and referral practice. Many additional cases will likely be managed in outpatient settings, requiring expanded provision, improved MRI, and strengthened cardiovascular prevention.

## Introduction

The World Health Organization’s 11th International Classification of Diseases (ICD-11) expanded the definition of stroke to include patients with neurological symptoms <24 hours if neuroimaging confirms a brain lesion [1]. ICD-10 required symptoms persisting ≥24 hours or resulting in death [1]. Our systematic review estimated that this change could increase stroke incidence by 4.8–10.5 per 100 000 person-years [2], and the Geneva Stroke Study reported an 18.3% increase [3].

Applying ICD-11 criteria to the population-based South London Stroke Register (SLSR), we observed a 6.8% increase in incidence [4]. These newly identified patients were a younger, predominantly male, White group with milder strokes and better outcomes [4]. While the national implications for UK health service planning remain unknown, understanding the distribution of the projected increase is crucial, as the Sentinel Stroke National Audit Programme (SSNAP) already provides a framework to assess service consequences in England, Wales, and Northern Ireland [5]. This study projects the impact of ICD-11 classification across the UK and examines its geographic distribution.

## Methods

We applied age- and sex-specific incidence rates from the SLSR (April 2022–April 2024) to UK Census populations using direct standardization. The SLSR is a population-based cohort study capturing first-ever strokes among residents of a defined multi-ethnic South London area [6]. Stroke was classified under both ICD-10 (symptoms ≥24 hours or death) and ICD-11 (ICD-10 cases, plus symptoms <24 hours with imaging-confirmed lesion).

Census 2021 data for England and Wales were obtained from the Office for National Statistics (ONS); Census 2021 data for Northern Ireland from the Northern Ireland Statistics and Research Agency; and Census 2022 data for Scotland from National Records of Scotland. Analyses were conducted at level 1, four UK countries; level 2, 33 health regions (seven NHS England regions, seven Welsh Local Health Boards, 14 Scottish health boards, and five Northern Ireland Health and Social Care Trusts); and level 3, 69 sub-regions (42 NHS Integrated Care Boards [ICB], seven Welsh Local Health Boards [LHB], 14 Scottish health boards, and six Northern Ireland counties).

Using the SLSR, stratum-specific incidence rates were calculated per 100 000 person-years across age- and sex-specific strata for the main analysis, and ethnicity for the sensitivity analysis (Table S1). Disaggregated data for age, sex, and ethnicity are unavailable at health board level in Scotland and Northern Ireland (de-anonymization risk), so the main analysis used age and sex standardization only. For England and Wales, a sensitivity analysis included ethnicity in the standardization to quantify the impact of omitting it. We also examined whether this impact varied by regional ethnic composition, using urban/rural classification as a contextual descriptor. The proportion of non-White population per ICB and LHB was derived from Census 2021 and correlated with the magnitude of overestimation using Pearson’s correlation coefficient. Urban/rural classification used the ONS Rural Urban Classification 2021, aggregated from Local Authority District to ICB level. A second sensitivity analysis examined missing data on symptom duration, using a conservative projection (missing data classified as stroke only under ICD-10) and a liberal projection (classified only under ICD-11).

Directly standardized rates (DSR) per 100 000 person-years and incidence rate ratios (IRR, ICD-11 vs ICD-10) with 95% confidence intervals were estimated using the Dobson method [7]; confidence intervals were derived from the Poisson distribution of observed counts. Ethical approval for the SLSR has been previously described [4].

## Results

**Figure 1.**
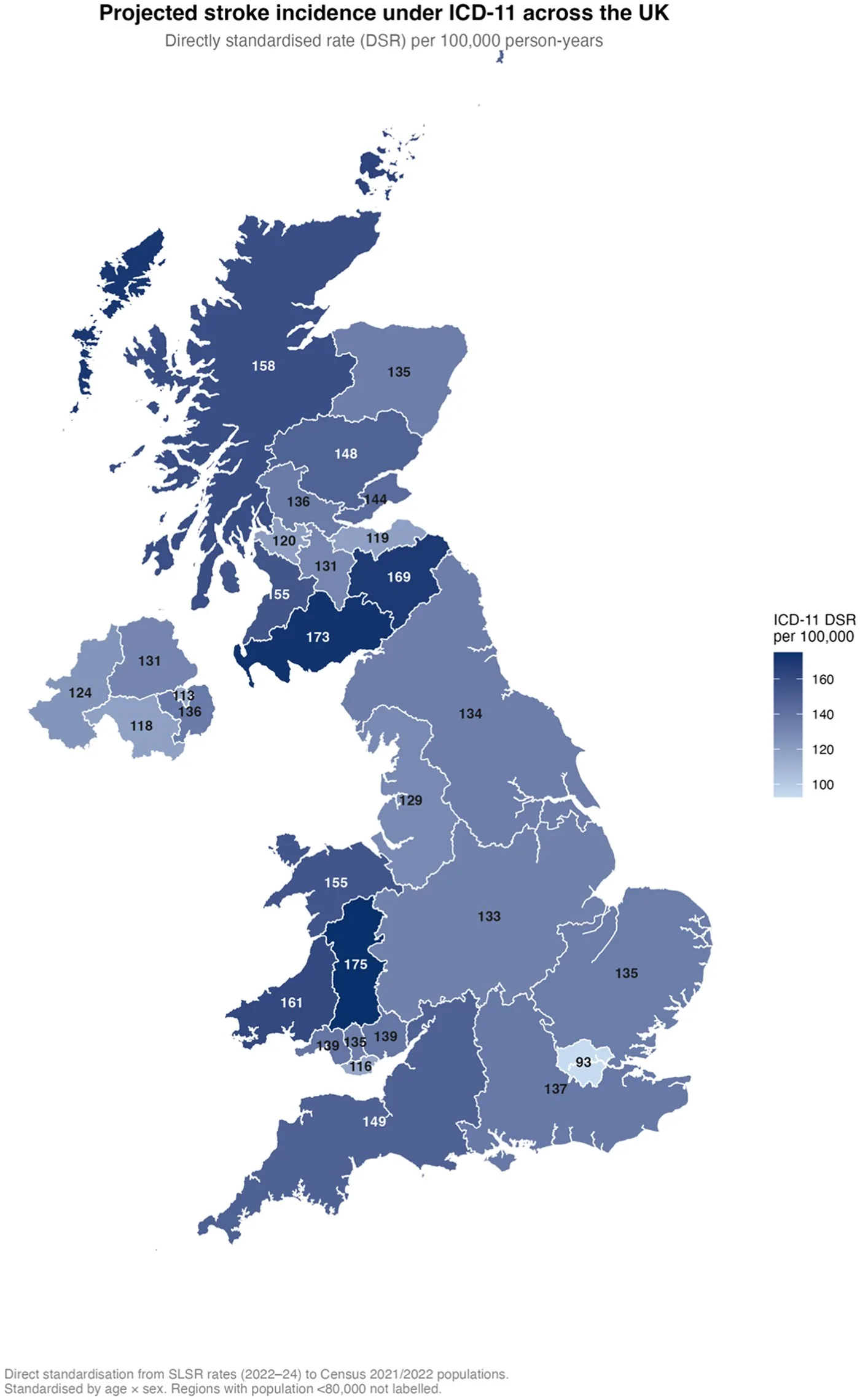
Directly standardized rate (DSR) per 100 000 person-years, standardized by age × sex. Source rates: South London Stroke Register, April 2022–April 2024 (*n* = 672 strokes). Census 2021/2022 populations. Regions with population <150 000 (Orkney, Shetland, Western Isles) were not labelled.

Across the UK, ICD-11 is projected to increase stroke incidence by ∼4.2% across all four nations (additional cases per year: UK = 2771; England = 2313; Scotland = 241; Wales = 142; Northern Ireland = 75; Table S2, Fig. 1). Projected ICD-11 rates were highest in Wales (142.6 per 100 000, 111.3–183.0) and the lowest in Northern Ireland (125.0, 97.6–160.4). The IRR was significant for England (1.04; 1.03–1.05) and Scotland (1.04; 1.005–1.08). This change was not uniform across the population but concentrated among males and those aged <65.

At health region level (level 2) and sub-regional level (level 3), DSRs ranged from 92.6 per 100 000 in London to 175.1 in Powys, Wales. Full results are available in Tables S3–S5 and an interactive dashboard at https://strokedashboard.org/evidence.php.

In the sensitivity analysis for England and Wales, adding ethnicity adjustment reduced projected DSRs by 10%–14%, with the smallest attenuation in London (−5% to −10%; Figs S1 and S2, Table S6). The magnitude of overestimation was inversely correlated with regional ethnic diversity (Pearson *r* = 0.61) and was not explained by urban/rural classification alone (Fig. S3). In the second sensitivity analysis, the conservative approach yielded a projected increase of 4.1% and the liberal approach 5.3%.

## Discussion

ICD-11 is projected to increase stroke incidence by ∼4.2% nationally, corresponding to an estimated 2771 additional cases per year. These figures are scenario estimates rather than forecasts of observed future incidence; whether the projected increase materializes will depend on local MRI availability, referral pathways, clinical coding practice, and outpatient diagnostic capacity. Under this uniform-rate assumption, the relative increase is consistent across regions, but projected absolute rates are overestimated in regions with a predominantly White population when ethnicity adjustment is omitted, as pooled rates reflect a more ethnically diverse population.

Our estimate is at the lower end of the range reported in our systematic review (4.8%–10.5%) [2] and below Geneva’s increase [3], where near-universal MRI was used. Applying SLSR-derived rates uniformly across the UK assumes imaging practice comparable to a well-resourced urban stroke service, unlikely to reflect national variation. In the SLSR, most new ICD-11 cases were detected by MRI [4]. Where MRI access is limited, particularly in rural areas [8], new ICD-11 strokes will be misclassified as TIA. If these projections do not materialize, neuroimaging and diagnostic pathways should be revised to guide imaging investment and stroke assessment.

The SLSR includes a higher proportion of Black Caribbean and Black African residents than most UK regions [9]. Applying pooled SLSR rates to regions with predominantly White populations inflates projected burden, explaining the reduction after ethnicity adjustment. This is a population-composition effect arising from the SLSR source population rather than a definitive correction of national burden; comparable adjustment is not available for Scotland and Northern Ireland, limiting UK-wide comparability. The pattern also reflects a clinical difference: ICD-11 mainly identifies younger, predominantly White patients with milder transient presentations whose infarcts are often missed on CT imaging, while Black Caribbean and Black African patients are less likely to present with milder, transitory symptoms, already being captured under ICD-10 [10]. They may also be less likely to seek medical attention for mild symptoms, given greater prehospital delays [S1]. Recent increases in stroke incidence in this cohort have been driven primarily by rising rates in Black ethnic groups, reflecting inequalities in cardiovascular risk rather than changes in case definition [9]. The projected 4.2% increase represents diagnostic reclassification of previously uncounted events, occurring alongside a sustained and substantial increase driven by ethnic and socioeconomic inequalities [9].

Ethnic and socioeconomic inequalities in stroke incidence exceed the effect attributable to reclassification and reflect differences in cardiovascular risk factor detection and management that a change in diagnostic criteria cannot modify [4, 9, S2]. The COVID-19 pandemic may have widened these inequalities [S3]. Analysis of 1.3 billion medication records across England, Scotland and Wales estimated 491 306 fewer individuals initiated antihypertensive treatment than expected in 2020–21, with lower post-pandemic growth among people with Black ethnicity [S3], and predicted more future strokes if these inequalities persist, consistent with our own findings [9]. These trends are independent of ICD-11 reclassification and likely to affect future incidence more than reclassification itself.

Many newly identified cases will be managed through outpatient pathways, outside SSNAP’s original scope [S4], risking gaps in quality assurance, missed diagnosis, and failure to initiate secondary prevention, leaving patients vulnerable to a predictable, more severe recurrent stroke. Implementation should be accompanied by expansion of outpatient capacity and audit coverage extended to outpatient care, including better symptom recognition, timely referrals, and increased staffing in outpatient and MRI services. SSNAP’s pilot of performance measures for minor stroke and TIA from April 2026 provides a framework to monitor progress.

As strengths, this analysis draws on population-based stroke surveillance from a well-characterized multi-ethnic cohort. Dual ICD-10 and ICD-11 classification was applied prospectively to the same cases, providing a robust basis for national projection rarely available from stroke registries. Its three-level geographic and ethnicity-adjusted analyses allow detailed assessment of variation. However, confidence intervals are wider for smaller health boards, and the SLSR overrepresents Black Caribbean and Black African communities relative to most UK regions. Further research must evaluate observed stroke incidence after ICD-11 implementation and, where the expected increase is not seen, prompt review of diagnostic and neuroimaging practices; costs, patient outcomes, and care-pathway impacts also warrant investigation.

## Conclusion

ICD-11 will increase recorded stroke incidence, and outpatient services should plan accordingly. The projected 4.2% rise is a scenario estimate that likely overestimates the impact of reclassification in regions with predominantly White populations, given the central assumption that South London rates apply uniformly across the UK and should be interpreted in the context of a broader increase in stroke incidence driven by inequalities in cardiovascular risk factors. This underlying trend is larger and potentially modifiable. Planning for ICD-11 should therefore be accompanied by strengthened and equitable primary prevention, improved access to neuroimaging and care, and appropriate outpatient monitoring and audit.

## Supplementary Material

ckag133_Supplementary_Data
