# Supplementary material for: National impact of ICD-11 stroke reclassification on projected incidence across the United Kingdom
Source: Eur J Public Health. 2026 Jul 22;36(4):ckag133. doi: 10.1093/eurpub/ckag133 (PMC13391154; doi:10.1093/eurpub/ckag133)
Supplement: ckag133_Supplementary_Data [file ckag133_supplementary_data.zip › ejph-2026-05-sr-0533-File005.docx]

## Supplementary Table S3. Projected stroke incidence by health region (Level 2, n=33)

| **Country** | **Region** | **Population** | **ICD-10 DSR (95% CI)** | **ICD-11 DSR (95% CI)** |
| --- | --- | --- | --- | --- |
| England | South West | 4,618,138 | 142.8 (110.7–184.5) | 148.5 (115.8–190.9) |
| England | South East | 7,112,129 | 131.9 (102.2–170.5) | 137.3 (107.0–176.6) |
| England | East of England | 5,227,690 | 130.0 (100.8–168.0) | 135.3 (105.5–174.0) |
| England | North East and Yorkshire | 6,710,349 | 129.1 (100.2–166.6) | 134.5 (105.0–172.7) |
| England | Midlands | 8,546,771 | 127.8 (99.1–165.1) | 133.1 (103.8–171.1) |
| England | North West | 5,598,678 | 123.9 (96.2–160.0) | 129.2 (100.8–165.8) |
| England | London | 6,901,651 | 88.7 (68.6–115.0) | 92.6 (72.0–119.4) |
| Northern Ireland | South Eastern | 285,981 | 130.7 (101.5–168.6) | 136.2 (106.3–174.8) |
| Northern Ireland | Northern | 371,783 | 125.8 (97.7–162.3) | 131.1 (102.4–168.3) |
| Northern Ireland | Western | 230,043 | 118.7 (92.3–153.0) | 124.0 (96.9–158.9) |
| Northern Ireland | Southern | 292,657 | 113.5 (88.1–146.5) | 118.5 (92.5–152.1) |
| Northern Ireland | Belfast | 287,623 | 108.6 (84.2–140.4) | 113.2 (88.2–145.6) |
| Scotland | Dumfries and Galloway | 120,514 | 166.0 (129.1–213.9) | 172.8 (135.1–221.5) |
| Scotland | Western Isles | 21,638 | 164.3 (127.9–211.5) | 171.0 (133.8–219.1) |
| Scotland | Borders | 96,140 | 162.6 (126.4–209.5) | 169.3 (132.3–217.0) |
| Scotland | Orkney | 18,076 | 157.4 (122.4–202.7) | 163.9 (128.2–210.0) |
| Scotland | Highland | 267,085 | 151.9 (118.1–195.6) | 158.2 (123.7–202.6) |
| Scotland | Ayrshire and Arran | 298,985 | 148.5 (115.5–191.2) | 154.7 (120.9–198.1) |
| Scotland | Shetland | 18,298 | 142.7 (110.9–183.9) | 148.7 (116.2–190.7) |
| Scotland | Tayside | 339,788 | 142.2 (110.4–183.5) | 148.0 (115.5–190.1) |
| Scotland | Fife | 301,818 | 137.9 (107.2–177.8) | 143.7 (112.3–184.2) |
| Scotland | Forth Valley | 245,553 | 130.8 (101.7–168.6) | 136.4 (106.6–174.8) |
| Scotland | Grampian | 470,565 | 129.2 (100.4–166.7) | 134.7 (105.2–172.8) |
| Scotland | Lanarkshire | 538,160 | 125.3 (97.5–161.4) | 130.7 (102.2–167.5) |
| Scotland | Greater Glasgow and Clyde | 963,692 | 115.4 (89.7–149.0) | 120.4 (94.0–154.5) |
| Scotland | Lothian | 740,430 | 114.0 (88.4–147.3) | 118.8 (92.7–152.7) |
| Wales | Powys | 109,688 | 168.2 (130.8–216.8) | 175.1 (136.8–224.4) |
| Wales | Hywel Dda | 311,007 | 155.1 (120.5–199.9) | 161.4 (126.1–207.0) |
| Wales | Betsi Cadwaladr | 552,951 | 148.5 (115.3–191.5) | 154.5 (120.7–198.3) |
| Wales | Swansea Bay | 306,657 | 133.4 (103.6–172.3) | 139.0 (108.4–178.5) |
| Wales | Aneurin Bevan | 465,951 | 133.0 (103.3–171.6) | 138.6 (108.3–177.9) |
| Wales | Cwm Taf Morgannwg | 350,775 | 129.6 (100.7–167.1) | 135.1 (105.6–173.3) |
| Wales | Cardiff and Vale | 392,846 | 111.2 (86.1–143.8) | 115.9 (90.3–149.0) |

Directly standardised rates (DSR) per 100,000 person-years (95% CI), standardised by age × sex. Regions sorted by country then descending ICD-11 DSR. IRR = incidence rate ratio (ICD-11 vs ICD-10).
