# Supplementary material for: National impact of ICD-11 stroke reclassification on projected incidence across the United Kingdom
Source: Eur J Public Health. 2026 Jul 22;36(4):ckag133. doi: 10.1093/eurpub/ckag133 (PMC13391154; doi:10.1093/eurpub/ckag133)
Supplement: ckag133_Supplementary_Data [file ckag133_supplementary_data.zip › ejph-2026-05-sr-0533-File007.docx]

## Supplementary Table S5. ICD-11 newly classified cases by ethnicity, SLSR source rates

| **Ethnicity** | **Person-years** | **ICD-10 cases** | **ICD-11 cases** | **ICD-10 rate/100k** | **ICD-11 rate/100k** | **% increase** | **IRR (95% CI)** |
| --- | --- | --- | --- | --- | --- | --- | --- |
| White | 565,831 | 283 | 306 | 50.0 | 54.1 | +8.1% | 1.081 (0.920–1.271) |
| Black Caribbean | 92,375 | 116 | 120 | 125.6 | 129.9 | +3.4% | 1.034 (0.801–1.335) |
| Black African | 146,954 | 157 | 161 | 106.8 | 109.6 | +2.5% | 1.025 (0.823–1.278) |
| Others | 219,341 | 90 | 91 | 41.0 | 41.5 | +1.1% | 1.011 (0.756–1.353) |
| **Total** | **1,024,501** | **646** | **678** | **63.1** | **66.2** | **+5.0%** | **1.050 (0.942–1.170)** |

Directly estimated from South London Stroke Register (April 2022–April 2024). Rates per 100,000 person-years. IRR = incidence rate ratio (ICD-11 vs ICD-10) with 95% confidence intervals. Ethnicity groups: White (includes White British, White Irish, White Other); Black Caribbean; Black African; Others (includes Asian, Mixed, Other ethnic groups).
