# Supplementary material for: National impact of ICD-11 stroke reclassification on projected incidence across the United Kingdom
Source: Eur J Public Health. 2026 Jul 22;36(4):ckag133. doi: 10.1093/eurpub/ckag133 (PMC13391154; doi:10.1093/eurpub/ckag133)
Supplement: ckag133_Supplementary_Data [file ckag133_supplementary_data.zip › ejph-2026-05-sr-0533-File004.docx]

## Supplementary Table S2. Projected stroke incidence under ICD-10 and ICD-11 by UK country

| **Country** | **Population** | **ICD-10 DSR (95% CI)** | **ICD-11 DSR (95% CI)** | **Additional cases/yr (%)** |
| --- | --- | --- | --- | --- |
| Wales | 2,489,879 | 136.9 (106.3–176.6) | 142.6 (111.3–183.0) | +142 (+4.2%) |
| Scotland | 4,435,519 | 129.0 (100.2–166.3) | 134.4 (105.0–172.4) | +241 (+4.2%) |
| England | 44,715,442 | 123.9 (96.1–160.2) | 129.1 (100.6–166.0) | +2,313 (+4.2%) |
| Northern Ireland | 1,468,110 | 119.8 (93.0–154.6) | 125.0 (97.6–160.4) | +75 (+4.3%) |
| **UK Total** | **53,108,950** | **124.4 (96.6–160.5)** | **130.0 (101.2–167.3)** | **+2,771 (+4.2%)** |

Directly standardised rates (DSR) per 100,000 person-years (95% CI), standardised by age × sex. Census 2021 (England, Wales, Northern Ireland) and Census 2022 (Scotland).
