# Supplementary material for: National impact of ICD-11 stroke reclassification on projected incidence across the United Kingdom
Source: Eur J Public Health. 2026 Jul 22;36(4):ckag133. doi: 10.1093/eurpub/ckag133 (PMC13391154; doi:10.1093/eurpub/ckag133)
Supplement: ckag133_Supplementary_Data [file ckag133_supplementary_data.zip › ejph-2026-05-sr-0533-File006.docx]

## Supplementary Table S4. Projected stroke incidence by health region (Level 3, n=69)

| **Country** | **Region** | **Population** | **ICD-10 DSR (95% CI)** | **ICD-11 DSR (95% CI)** |
| --- | --- | --- | --- | --- |
| **England** | NHS Dorset | 640,43 | 158.6 (122.9–205.0) | 164.7 (128.3–211.9) |
| **England** | NHS Somerset | 461,849 | 156.1 (121.1–201.5) | 162.3 (126.6–208.5) |
| **England** | NHS Cornwall and the Isles of Scilly | 467,256 | 155.1 (120.5–200.0) | 161.4 (126.1–207.1) |
| **England** | NHS Norfolk and Waveney | 843,736 | 153.7 (119.2–198.5) | 159.8 (124.6–205.4) |
| **England** | NHS Devon | 993,856 | 151.6 (117.6–195.8) | 157.7 (123.0–202.6) |
| **England** | NHS Herefordshire and Worcestershire | 638,929 | 149.1 (115.8–192.5) | 155.2 (121.1–199.3) |
| **England** | NHS Lincolnshire | 624,106 | 146.9 (114.1–189.6) | 153.0 (119.4–196.4) |
| **England** | NHS Suffolk and North East Essex | 792,628 | 145.7 (113.0–188.2) | 151.6 (118.2–194.8) |
| **England** | NHS Sussex | 1,375,800 | 144.7 (112.2–187.1) | 150.4 (117.2–193.5) |
| **England** | NHS Shropshire, Telford and Wrekin | 409,086 | 144.3 (112.1–186.2) | 150.3 (117.3–192.9) |
| **England** | NHS Humber and North Yorkshire | 1,370,652 | 142.3 (110.5–183.6) | 148.1 (115.6–190.1) |
| **England** | NHS Gloucestershire | 517,65 | 141.2 (109.5–182.3) | 146.9 (114.6–188.7) |
| **England** | NHS Staffordshire and Stoke-on-Trent | 907,152 | 136.2 (105.8–175.7) | 141.9 (110.8–182.1) |
| **England** | NHS Hampshire and Isle of Wight | 1,465,746 | 136.3 (105.7–176.2) | 141.9 (110.6–182.4) |
| **England** | NHS Lancashire and South Cumbria | 1,366,532 | 135.4 (105.1–174.7) | 141.1 (110.1–181.0) |
| **England** | NHS Derby and Derbyshire | 845,762 | 134.9 (104.8–174.1) | 140.6 (109.8–180.4) |
| **England** | NHS North East and North Cumbria | 2,382,311 | 133.9 (104.0–172.6) | 139.5 (109.0–178.9) |
| **England** | NHS Bath and North East Somerset, Swindon and Wiltshire | 751,43 | 132.2 (102.5–170.9) | 137.7 (107.3–177.0) |
| **England** | NHS Surrey Heartlands | 826,165 | 131.9 (102.1–170.7) | 137.1 (106.7–176.6) |
| **England** | NHS Kent and Medway | 1,455,694 | 131.6 (102.1–169.9) | 137.0 (106.9–176.0) |
| **England** | NHS Cheshire and Merseyside | 2,018,485 | 130.7 (101.5–168.7) | 136.2 (106.3–174.8) |
| **England** | NHS Mid and South Essex | 941,464 | 130.2 (101.0–168.2) | 135.5 (105.7–174.2) |
| **England** | NHS Coventry and Warwickshire | 746,222 | 123.9 (96.1–160.2) | 129.1 (100.6–166.0) |
| **England** | NHS South Yorkshire | 1,092,016 | 123.4 (95.8–159.4) | 128.7 (100.4–165.3) |
| **England** | NHS Nottingham and Nottinghamshire | 919,259 | 123.0 (95.4–158.9) | 128.2 (100.0–164.7) |
| **England** | NHS Hertfordshire and West Essex | 1,163,110 | 122.5 (94.9–158.6) | 127.5 (99.3–164.2) |
| **England** | NHS Buckinghamshire, Oxfordshire and Berkshire West | 1,398,684 | 122.2 (94.7–158.1) | 127.3 (99.1–163.8) |
| **England** | NHS Black Country | 929,377 | 121.4 (94.1–157.0) | 126.5 (98.6–162.7) |
| **England** | NHS Leicester, Leicestershire and Rutland | 885,914 | 121.0 (93.8–156.3) | 126.1 (98.3–162.0) |
| **England** | NHS Northamptonshire | 614,233 | 119.7 (92.9–154.5) | 124.8 (97.4–160.3) |
| **England** | NHS Cambridgeshire and Peterborough | 723,39 | 119.2 (92.4–154.2) | 124.2 (96.8–159.8) |
| **England** | NHS West Yorkshire | 1,865,349 | 116.6 (90.4–150.6) | 121.5 (94.8–156.2) |
| **England** | NHS Frimley | 590,046 | 114.9 (89.0–148.7) | 119.7 (93.2–154.1) |
| **England** | NHS Bristol, North Somerset and South Gloucestershire | 785,688 | 114.7 (88.8–148.5) | 119.5 (93.0–153.9) |
| **England** | NHS Greater Manchester | 2,213,670 | 110.6 (85.8–142.9) | 115.5 (90.1–148.3) |
| **England** | NHS Bedfordshire, Luton and Milton Keynes | 763,377 | 108.8 (84.4–140.7) | 113.6 (88.5–146.0) |
| **England** | NHS Birmingham and Solihull | 1,026,775 | 106.6 (82.5–138.0) | 111.1 (86.4–143.1) |
| **England** | NHS South West London | 1,180,100 | 96.7 (74.8–125.3) | 100.9 (78.5–130.0) |
| **England** | NHS North West London | 1,657,963 | 92.1 (71.2–119.4) | 96.1 (74.8–123.9) |
| **England** | NHS North Central London | 1,111,298 | 91.2 (70.5–118.3) | 95.2 (74.0–122.8) |
| **England** | NHS South East London | 1,419,156 | 88.4 (68.3–114.6) | 92.2 (71.7–118.9) |
| **England** | NHS North East London | 1,533,161 | 77.3 (59.7–100.5) | 80.9 (62.8–104.5) |
| **Northern Ireland** | Fermanagh | 48,757 | 131.5 (102.2–169.4) | 137.2 (107.2–175.8) |
| **Northern Ireland** | Down | 432,419 | 128.2 (99.5–165.4) | 133.6 (104.3–171.5) |
| **Northern Ireland** | Derry/Londonderry | 192,578 | 117.0 (91.0–150.9) | 122.1 (95.5–156.6) |
| **Northern Ireland** | Antrim | 507,875 | 116.2 (90.1–150.0) | 121.1 (94.5–155.6) |
| **Northern Ireland** | Tyrone | 141,592 | 114.8 (89.1–148.2) | 119.8 (93.5–153.8) |
| **Northern Ireland** | Armagh | 144,897 | 112.5 (87.3–145.2) | 117.4 (91.6–150.7) |
| **Scotland** | Dumfries and Galloway | 120,514 | 166.0 (129.1–213.9) | 172.8 (135.1–221.5) |
| **Scotland** | Western Isles | 21,638 | 164.3 (127.9–211.5) | 171.0 (133.8–219.1) |
| **Scotland** | Borders | 96,14 | 162.6 (126.4–209.5) | 169.3 (132.3–217.0) |
| **Scotland** | Orkney | 18,076 | 157.4 (122.4–202.7) | 163.9 (128.2–210.0) |
| **Scotland** | Highland | 267,085 | 151.9 (118.1–195.6) | 158.2 (123.7–202.6) |
| **Scotland** | Ayrshire and Arran | 298,985 | 148.5 (115.5–191.2) | 154.7 (120.9–198.1) |
| **Scotland** | Shetland | 18,298 | 142.7 (110.9–183.9) | 148.7 (116.2–190.7) |
| **Scotland** | Tayside | 339,788 | 142.2 (110.4–183.5) | 148.0 (115.5–190.1) |
| **Scotland** | Fife | 301,818 | 137.9 (107.2–177.8) | 143.7 (112.3–184.2) |
| **Scotland** | Forth Valley | 245,553 | 130.8 (101.7–168.6) | 136.4 (106.6–174.8) |
| **Scotland** | Grampian | 470,565 | 129.2 (100.4–166.7) | 134.7 (105.2–172.8) |
| **Scotland** | Lanarkshire | 538,16 | 125.3 (97.5–161.4) | 130.7 (102.2–167.5) |
| **Scotland** | Greater Glasgow and Clyde | 963,692 | 115.4 (89.7–149.0) | 120.4 (94.0–154.5) |
| **Scotland** | Lothian | 740,43 | 114.0 (88.4–147.3) | 118.8 (92.7–152.7) |
| **Wales** | Powys | 109,688 | 168.2 (130.8–216.8) | 175.1 (136.8–224.4) |
| **Wales** | Hywel Dda | 311,007 | 155.1 (120.5–199.9) | 161.4 (126.1–207.0) |
| **Wales** | Betsi Cadwaladr | 552,951 | 148.5 (115.3–191.5) | 154.5 (120.7–198.3) |
| **Wales** | Swansea Bay | 306,657 | 133.4 (103.6–172.3) | 139.0 (108.4–178.5) |
| **Wales** | Aneurin Bevan | 465,951 | 133.0 (103.3–171.6) | 138.6 (108.3–177.9) |
| **Wales** | Cwm Taf Morgannwg | 350,775 | 129.6 (100.7–167.1) | 135.1 (105.6–173.3) |
| **Wales** | Cardiff and Vale | 392,846 | 111.2 (86.1–143.8) | 115.9 (90.3–149.0) |

Directly standardised rates (DSR) per 100,000 person-years (95% CI), standardised by age × sex. Regions sorted by country then descending ICD-11 DSR. IRR = incidence rate ratio (ICD-11 vs ICD-10).
