# Supplementary material for: National impact of ICD-11 stroke reclassification on projected incidence across the United Kingdom
Source: Eur J Public Health. 2026 Jul 22;36(4):ckag133. doi: 10.1093/eurpub/ckag133 (PMC13391154; doi:10.1093/eurpub/ckag133)
Supplement: ckag133_Supplementary_Data [file ckag133_supplementary_data.zip › ejph-2026-05-sr-0533-File008.docx]

## Supplementary Table S6. Impact of ethnicity adjustment on projected stroke incidence under ICD-10 and ICD-11 by health region (England and Wales)

| **Country** | **Region** | **ICD-10 DSR (age × sex)** | **ICD-11 DSR (age × sex)** | **% increase (age × sex)** | **ICD-10 DSR (age × sex × eth)** | **ICD-11 DSR (age × sex × eth)** | **% increase (age × sex × eth)** | **% change in ICD-11 DSR level** |
| --- | --- | --- | --- | --- | --- | --- | --- | --- |
| England | South West | 142,8 | 148,5 | 4 | 121,6 | 129,4 | 6,4 | -12,9 |
| England | South East | 131,9 | 137,3 | 4,1 | 112,2 | 119,2 | 6,3 | -13,1 |
| England | East of England | 130 | 135,3 | 4,1 | 111 | 118 | 6,3 | -12,8 |
| England | North East and Yorkshire | 129,1 | 134,5 | 4,2 | 108,9 | 116 | 6,5 | -13,7 |
| England | Midlands | 127,8 | 133,1 | 4,2 | 109,2 | 116 | 6,2 | -12,9 |
| England | North West | 123,9 | 129,2 | 4,2 | 104,8 | 111,6 | 6,5 | -13,6 |
| England | London | 88,7 | 92,6 | 4,4 | 79,9 | 83,7 | 4,8 | -9,6 |
| Wales | Powys Teaching Health Board | 168,2 | 175,1 | 4,1 | 143,3 | 152,7 | 6,6 | -12,8 |
| Wales | Hywel Dda University Health Board | 155,1 | 161,4 | 4,1 | 131,5 | 140,1 | 6,6 | -13,2 |
| Wales | Betsi Cadwaladr University Health Board | 148,5 | 154,5 | 4,1 | 125,7 | 134 | 6,6 | -13,3 |
| Wales | Swansea Bay University Health Board | 133,4 | 139 | 4,1 | 112,7 | 120,1 | 6,6 | -13,6 |
| Wales | Aneurin Bevan University Health Board | 133 | 138,6 | 4,2 | 112 | 119,5 | 6,7 | -13,8 |
| Wales | Cwm Taf Morgannwg University Health Board | 129,6 | 135,1 | 4,3 | 108,6 | 116,1 | 6,9 | -14,1 |
| Wales | Cardiff and Vale University Health Board | 111,2 | 115,9 | 4,2 | 94,3 | 100,2 | 6,4 | -13,5 |

DSR per 100,000 person-years derived from SLSR rates (April 2022–April 2024) applied to Census 2021 populations. Age × sex standardisation uses pooled SLSR rates (main analysis); age × sex × ethnicity standardisation uses ethnicity-specific SLSR rates (sensitivity analysis). % increase = (ICD-11 − ICD-10) / ICD-10 × 100. % change in ICD-11 DSR level = (age×sex×ethnicity DSR − age×sex DSR) / age×sex DSR × 100; negative values indicate that pooled SLSR rates overestimate absolute stroke burden relative to ethnicity-adjusted estimates in predominantly White regions.
