# Supplementary material for: National impact of ICD-11 stroke reclassification on projected incidence across the United Kingdom
Source: Eur J Public Health. 2026 Jul 22;36(4):ckag133. doi: 10.1093/eurpub/ckag133 (PMC13391154; doi:10.1093/eurpub/ckag133)
Supplement: ckag133_Supplementary_Data [file ckag133_supplementary_data.zip › ejph-2026-05-sr-0533-File003.docx]

## Supplementary Table S1. Stratum-specific stroke incidence rates from the South London Stroke Register (SLSR), April 2022–April 2024

| **Age band** | **Sex** | **Ethnicity** | **n**  **(ICD-10)** | **n**  **(ICD-11)** | **Person-**  **years** | **ICD-10 rate**  **(95% CI)** | **ICD-11 rate**  **(95% CI)** |
| --- | --- | --- | --- | --- | --- | --- | --- |
| 18–44 | Female | Black African | 10 | 11 | 43,222 | 23.1 (12.6–42.6) | 25.4 (14.2–45.6) |
| 45–54 | Female | Black African | 14 | 14 | 17,978 | 77.9 (46.4–130.7) | 77.9 (46.4–130.7) |
| 55–64 | Female | Black African | 15 | 15 | 13,249 | 113.2 (68.6–186.8) | 113.2 (68.6–186.8) |
| 65–74 | Female | Black African | 16 | 16 | 4,318 | 370.5 (228.1–601.9) | 370.5 (228.1–601.9) |
| 75–84 | Female | Black African | 14 | 14 | 2,307 | 606.8 (361.5–1018.7) | 606.8 (361.5–1018.7) |
| 85+ | Female | Black African | 3 | 3 | 569 | 526.9 (179.2–1549.2) | 526.9 (179.2–1549.2) |
| 18–44 | Female | Black Caribbean | 5 | 5 | 26,599 | 18.8 (8.0–44.0) | 18.8 (8.0–44.0) |
| 45–54 | Female | Black Caribbean | 4 | 4 | 8,760 | 45.7 (17.8–117.4) | 45.7 (17.8–117.4) |
| 55–64 | Female | Black Caribbean | 18 | 19 | 10,610 | 169.6 (107.3–268.2) | 179.1 (114.6–279.7) |
| 65–74 | Female | Black Caribbean | 13 | 13 | 3,265 | 398.2 (232.7–681.4) | 398.2 (232.7–681.4) |
| 75–84 | Female | Black Caribbean | 16 | 16 | 2,224 | 719.4 (442.8–1168.6) | 719.4 (442.8–1168.6) |
| 85+ | Female | Black Caribbean | 10 | 10 | 1,414 | 707.4 (384.3–1302.3) | 707.4 (384.3–1302.3) |
| 18–44 | Female | Others | 8 | 8 | 79,005 | 10.1 (5.1–19.9) | 10.1 (5.1–19.9) |
| 45–54 | Female | Others | 6 | 6 | 18,486 | 32.5 (14.9–70.8) | 32.5 (14.9–70.8) |
| 55–64 | Female | Others | 8 | 8 | 11,507 | 69.5 (35.2–137.2) | 69.5 (35.2–137.2) |
| 65–74 | Female | Others | 11 | 11 | 5,198 | 211.6 (118.2–379.0) | 211.6 (118.2–379.0) |
| 75–84 | Female | Others | 8 | 8 | 2,840 | 281.7 (142.7–555.9) | 281.7 (142.7–555.9) |
| 85+ | Female | Others | 6 | 6 | 765 | 784.3 (359.5–1711.3) | 784.3 (359.5–1711.3) |
| 18–44 | Female | White | 6 | 6 | 189,580 | 3.2 (1.5–6.9) | 3.2 (1.5–6.9) |
| 45–54 | Female | White | 10 | 10 | 30,607 | 32.7 (17.7–60.1) | 32.7 (17.7–60.1) |
| 55–64 | Female | White | 16 | 16 | 27,606 | 58.0 (35.7–94.2) | 58.0 (35.7–94.2) |
| 65–74 | Female | White | 25 | 27 | 17,201 | 145.3 (98.4–214.6) | 157.0 (107.9–228.4) |
| 75–84 | Female | White | 30 | 32 | 8,528 | 351.8 (246.4–502.2) | 375.2 (265.8–529.7) |
| 85+ | Female | White | 35 | 35 | 4,343 | 805.8 (579.4–1120.6) | 805.8 (579.4–1120.6) |
| 18–44 | Male | Black African | 5 | 6 | 34,787 | 14.4 (6.1–33.6) | 17.2 (7.9–37.6) |
| 45–54 | Male | Black African | 14 | 15 | 13,225 | 105.9 (63.1–177.7) | 113.4 (68.7–187.1) |
| 55–64 | Male | Black African | 33 | 34 | 12,008 | 274.8 (195.7–385.9) | 283.1 (202.6–395.7) |
| 65–74 | Male | Black African | 21 | 21 | 3,782 | 555.2 (363.2–848.8) | 555.2 (363.2–848.8) |
| 75–84 | Male | Black African | 9 | 9 | 1,232 | 730.6 (384.4–1388.7) | 730.6 (384.4–1388.7) |
| 85+ | Male | Black African | 3 | 3 | 277 | 1084.6 (368.9–3189.1) | 1084.6 (368.9–3189.1) |
| 18–44 | Male | Black Caribbean | 2 | 2 | 21,097 | 9.5 (2.6–34.6) | 9.5 (2.6–34.6) |
| 45–54 | Male | Black Caribbean | 4 | 4 | 7,050 | 56.7 (22.1–145.9) | 56.7 (22.1–145.9) |
| 55–64 | Male | Black Caribbean | 16 | 17 | 6,958 | 230.0 (141.6–373.6) | 244.3 (152.6–391.3) |
| 65–74 | Male | Black Caribbean | 11 | 13 | 2,137 | 514.7 (287.4–921.7) | 608.3 (355.5–1040.8) |
| 75–84 | Male | Black Caribbean | 5 | 5 | 1,526 | 327.7 (140.0–767.2) | 327.7 (140.0–767.2) |
| 85+ | Male | Black Caribbean | 12 | 12 | 737 | 1628.7 (931.7–2847.0) | 1628.7 (931.7–2847.0) |
| 18–44 | Male | Others | 5 | 6 | 67,499 | 7.4 (3.2–17.3) | 8.9 (4.1–19.4) |
| 45–54 | Male | Others | 9 | 9 | 16,678 | 54.0 (28.4–102.6) | 54.0 (28.4–102.6) |
| 55–64 | Male | Others | 16 | 16 | 11,036 | 145.0 (89.2–235.5) | 145.0 (89.2–235.5) |
| 65–74 | Male | Others | 7 | 7 | 4,034 | 173.5 (84.1–358.2) | 173.5 (84.1–358.2) |
| 75–84 | Male | Others | 4 | 4 | 1,777 | 225.1 (87.6–579.0) | 225.1 (87.6–579.0) |
| 85+ | Male | Others | 2 | 2 | 517 | 387.1 (106.2–1411.7) | 387.1 (106.2–1411.7) |
| 18–44 | Male | White | 7 | 11 | 190,304 | 3.7 (1.8–7.6) | 5.8 (3.2–10.4) |
| 45–54 | Male | White | 19 | 21 | 38,579 | 49.2 (31.5–76.9) | 54.4 (35.6–83.2) |
| 55–64 | Male | White | 36 | 42 | 31,734 | 113.4 (82.0–157.0) | 132.4 (97.9–178.9) |
| 65–74 | Male | White | 43 | 46 | 17,912 | 240.1 (178.2–323.3) | 256.8 (192.6–342.5) |
| 75–84 | Male | White | 41 | 45 | 7,268 | 564.1 (415.8–765.2) | 619.1 (462.7–828.4) |
| 85+ | Male | White | 15 | 15 | 2,167 | 692.1 (419.5–1142.1) | 692.1 (419.5–1142.1) |
