# Supplementary figures and images for: National impact of ICD-11 stroke reclassification on projected incidence across the United Kingdom
Source: Eur J Public Health. 2026 Jul 22;36(4):ckag133. doi: 10.1093/eurpub/ckag133 (PMC13391154; doi:10.1093/eurpub/ckag133)

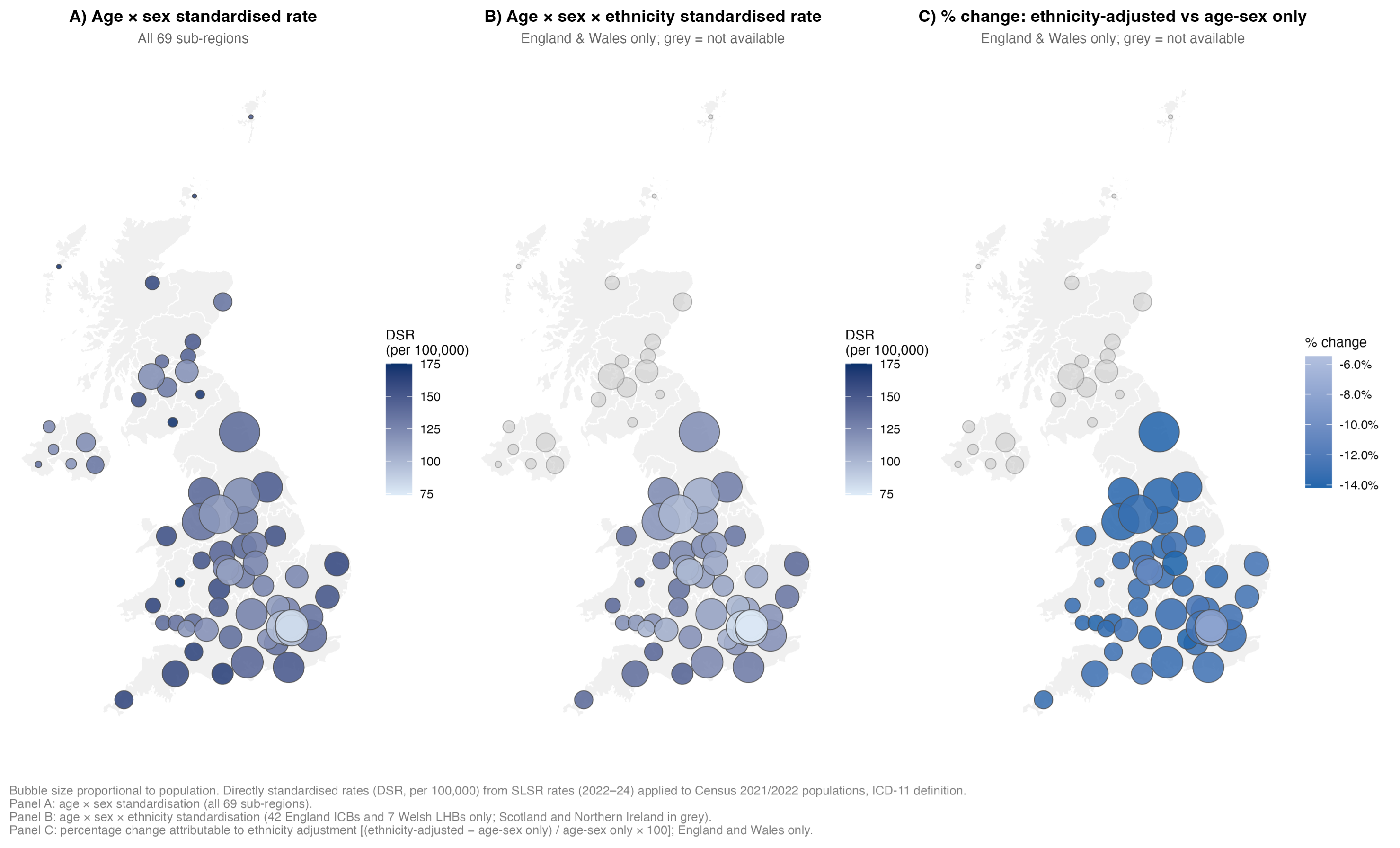

Supplement: ckag133_Supplementary_Data [file ckag133_supplementary_data.zip › ejph-2026-05-sr-0533-File010.tiff]

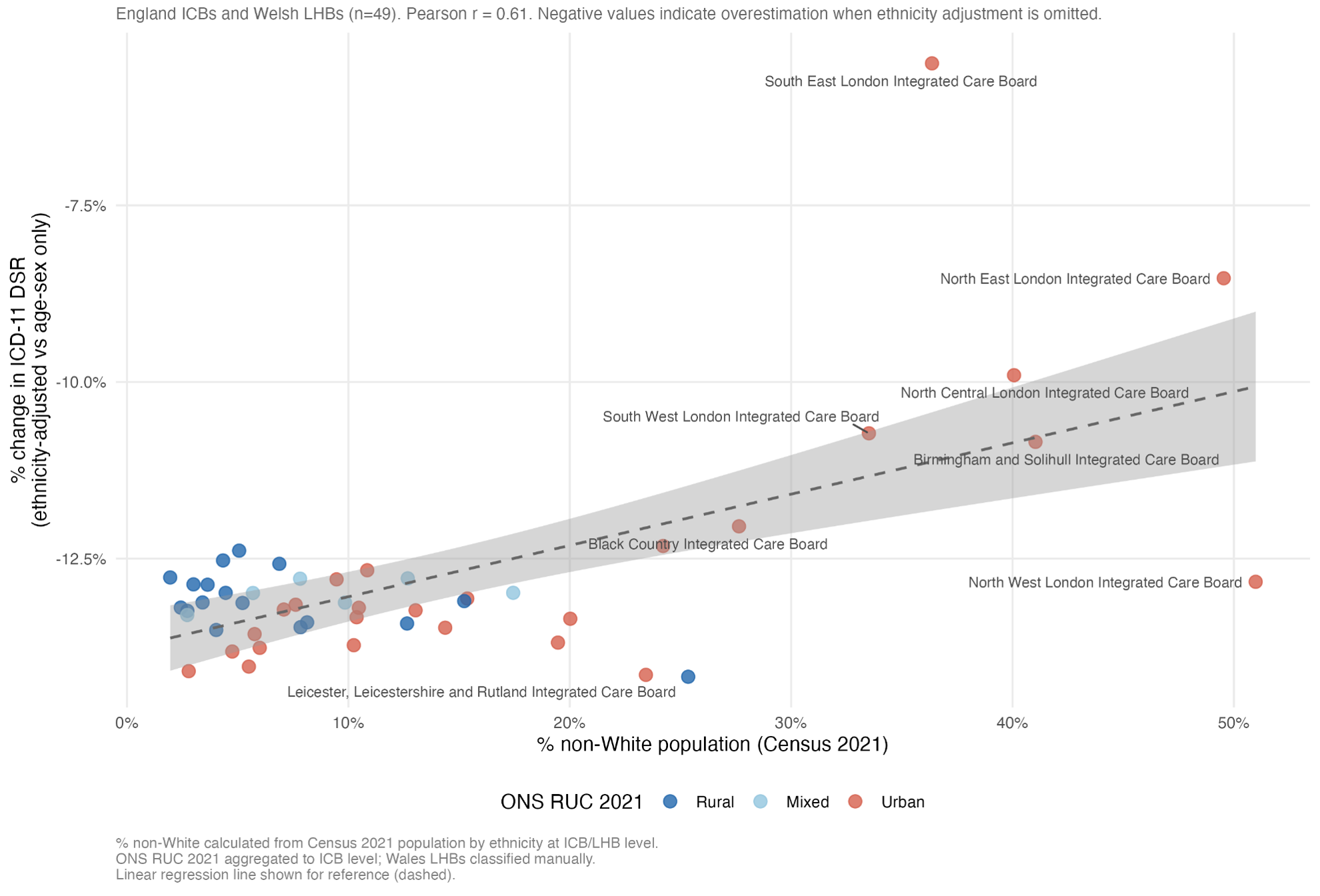

Supplement: ckag133_Supplementary_Data [file ckag133_supplementary_data.zip › ejph-2026-05-sr-0533-File011.tiff]

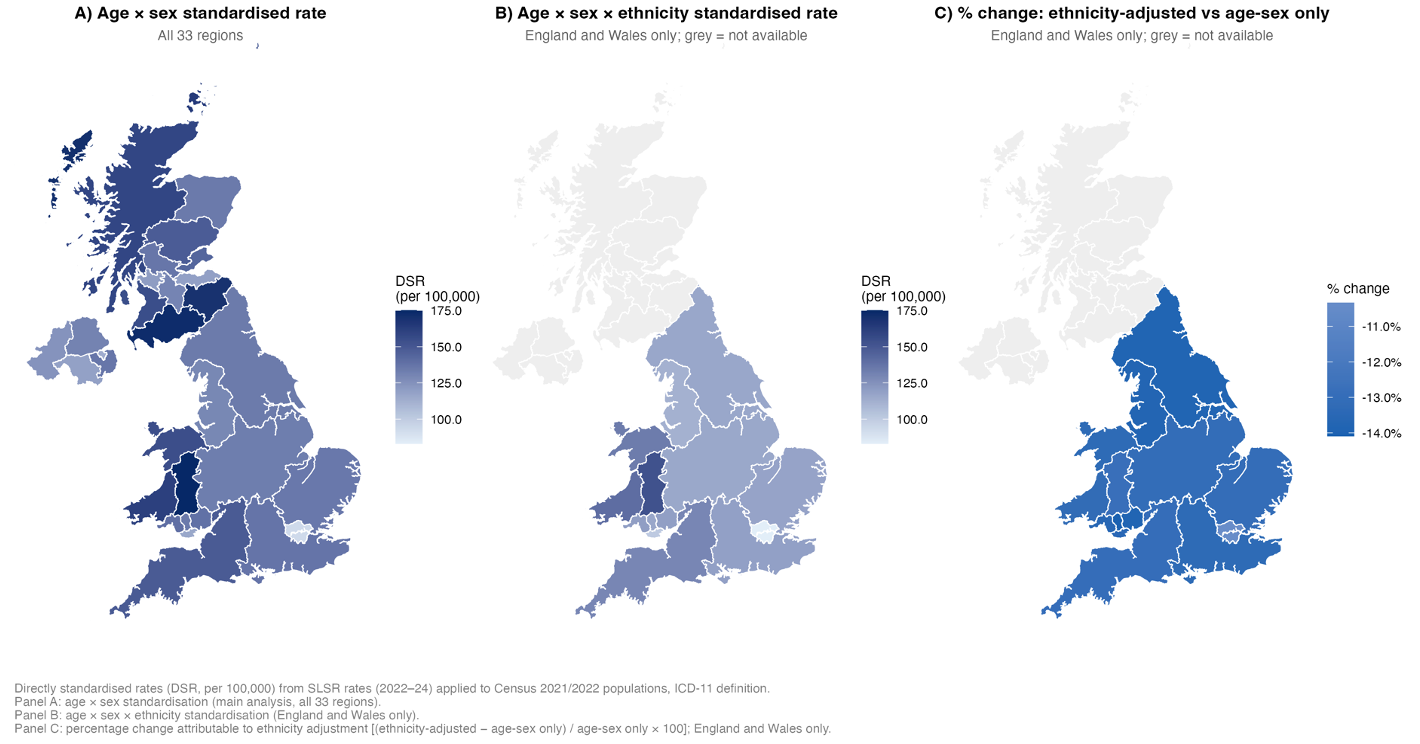

Supplement: ckag133_Supplementary_Data [file ckag133_supplementary_data.zip › ejph-2026-05-sr-0533-File009.tiff]
